# Analysis of the genome of a Pseudomonas monsensis isolate that produces the antifungal dipeptide maculosin

**DOI:** 10.1099/acmi.0.001229.v3

**Published:** 2026-09-15

**Authors:** Priyanka Sharma, Alexander D. H. Kingdon, Jack K. Bradley, Ellie Allman, Song Ang, Matthew Pye, Angus M. O’Ferrall, Amy McLeman, Claudia McKeown, W. David Hong, Adam P. Roberts

**Affiliations:** 1 Department of Tropical Disease Biology, Centre for Drugs and Diagnostics, Liverpool School of Tropical Medicine, Liverpool, L3 5QA, UK; 2 Department of Chemistry, University of Liverpool, Liverpool, L3 5QA, UK; 3 School of Pharmacy and Food Engineering, Guangdong Provincial Key Laboratory of Large Animal Models for Biomedicine, Wuyi University, Jiangmen 529020, PR China

**Keywords:** biosynthetic gene clusters, *Candida albicans*, cyclo(Pro-Tyr), genome sequencing, high-performance liquid chromatography, Swab and Send, citizen science

## Abstract

*Candida* species have been attributed to causing ~647,000 deaths annually. *Candida albicans* has been identified by the WHO as a priority pathogen for targeted antifungal drug development. To this end, we have screened microbial strains from the public outreach project Swab and Send for anti-*Candida* activity. This process identified *Pseudomonas* sp. SS1954.14, which displayed potent activity against *C. albicans*, both on agar and in a cell-free supernatant assay. Whole-genome sequence analysis identified this strain as *Pseudomonas monsensis*. It contains several biosynthetic gene clusters, suggesting that it can produce hydrogen cyanide, lokisin, pyoverdine and colicin/carocin, which may contribute to the observed antifungal activity. However, an active compound was separated by preparative high-performance liquid chromatography and identified through high-resolution mass spectrometry as maculosin [cyclo(Pro-Tyr)]. This cyclic dipeptide has previously been found to possess antifungal activity, but the exact biosynthetic mechanism remains undetermined. Reporting this genome alongside the associated evidence of maculosin production represents a valuable resource for biosynthetic investigations.

## Data Summary

 The genomics data are available as part of BioProject PRJNA1161700. The assembled and annotated genome has been deposited in GenBank under accession no. JBXOEP000000000. The version described in this paper is version JBXOEP010000000. The raw MS data have been deposited to MassIVE under dataset ID MSV000102665, accessible at doi: 10.25345 /C5T727W6K.

## Introduction

Fungal infections represent a growing public health concern, with ~6.5 million annual invasive fungal infections, resulting in ~2.5 million attributable deaths [1]. When focusing on invasive candidiasis and *Candida* bloodstream infections, these account for 1,565,000 infections and ~647,000 attributable deaths [1]. The majority of candidiasis infections are caused by six species: *Candida albicans*, *Candida auris*, *Candida tropicalis*, *Candida parapsilosis*, *Candida krusei* and *Nakaseomyces glabratus* (formerly *Candida glabrata*) [2, 3]. *C. albicans* is the most common cause of candidiasis, responsible for ~65% of cases [2].

There are three main classes of antifungals used clinically: azoles, echinocandins and polyenes. These classes target either the fungal cell membrane (azoles and polyenes) or the cell wall (echinocandins). Azoles inhibit ergosterol synthesis, echinocandins inhibit *β*-(1,3)-d-glucan synthesis, and polyenes bind to ergosterol and induce pore formation [4]. Resistance in *C. albicans* is uncommon, but it has been increasing to all three of these classes of antifungals [5–7]. In particular, resistance to azoles has been increasing through mutations within the *ERG11* gene [8–10]. The World Health Organization has highlighted that the current pipeline of antifungal drugs in clinical and preclinical development is insufficient to meet future clinical need [11]. Only four drug candidates currently undergoing clinical evaluation have activity against *C. albicans*, and only one is being trialled for use against *C. albicans* [11]. The discovery and development of additional antifungal compounds are required to address this growing issue.

Swab and Send is a citizen science antimicrobial discovery project [12] that involves screening microbes for antimicrobial activity against bacterial and fungal isolates of interest. We identified a species of *Pseudomonas*, designated as SS1954.14, which was cultured from a Winogradsky column and displayed potent antifungal activity against *C. albicans*. Genome analysis revealed several possible antifungal compounds that were likely encoded. However, purification from the antifungal cell-free supernatant (CFS) of the strain, by activity tracking, identified the cyclic dipeptide natural product maculosin [cyclo(Pro-Tyr)].

## Methods

### Bacterial isolation

Sterile TRANSWAB Amies Charcoal swabs (MWE) were shared as part of Swab and Send, and once a sample of interest was swabbed, the swabs were stored in Amies charcoal agar at room temperature for up to 3 days and then at 4 °C thereafter. The swabs were spread onto brain heart infusion (BHI) agar and incubated at room temperature for 2 days until colonies developed. Those with distinct morphologies were picked into BHI broth (100 µl) and incubated statically at room temperature for a further 2 days. Strains were stored by adding 40% glycerol in BHI broth (100 µl) to each well, resulting in a final glycerol volume of 20% (v/v), followed by freezing at –70 °C.

### Agar testing

The isolate was screened on agar on top of a lawn of test strains *C. albicans* or *Escherichia coli*. The lawn was prepared by diluting an overnight culture of the test strains to an OD_600_ 4.0 (*C. albicans*) or 0.15 (*E. coli*) in BHI broth and spreading it with a sterile swab across the surface of a BHI agar plate. The isolates were stamped out using a 96-pin replicator from a glycerol stock, within 30 min of preparing the lawn, transferring ~5 µl on top of the lawn. The plate was incubated at room temperature for 2 days until clear growth of the test organism was observed, and any zones of growth inhibition could be recorded.

### Cell-free supernatant **a**ssay

Test organisms *E. coli* (NCTC 86) and *C. albicans* (ATCC 90028) were plated onto BHI agar and incubated at room temperature for 48 h. Test organism colonies were then selected and used to inoculate 8 ml of BHI broth and grown overnight (18–20 h) at 180 r.p.m., 37 °C. *Pseudomonas* sp. SS1954.14 was transferred to BHI broth and allowed to grow overnight (18–20 h) at 37 °C. An aliquot of overnight culture (1 ml) was centrifuged at 14,800 ***g*** for 1 min. Supernatant was filtered through a 0.22 µm membrane to remove any test organism cells. Samples of the filtered supernatant (100 µl per well) were added to clear 96-well plates, alongside 100 µl of BHI broth in the control wells. The OD_600_ of the test strain was normalized to ~0.1 through the addition of 80 µl of the overnight test organism’s culture to 4.5 ml sterile BHI and adjusted to obtain a 0.5 McFarland standard (OD_600_ ~0.1). For *C. albicans*, 100 µl of the OD_600_ 0.1 test organism was added to 4,900 µl (1 : 50) of BHI broth to obtain OD_600_ ~0.002. For *E. coli*, 1 µl of the OD_600_ 0.1 test organism was added to 4,999 µl (1 : 5,000) of BHI broth to obtain OD_600_ ~0.00002. Then, 100 µl of OD_600_ 0.002 (*Candida*) or 0.00002 (*E. coli*) solution was added to the supernatant and control wells, becoming OD_600_ 0.001 (*C. albicans*) or 0.00001 (*E. coli*). The OD_600_ was then monitored every 10 min for 19 h using a CLARIOstar plus microplate reader (BMG Labtech).

### Whole-genome sequencing

Genus was identified prior to sending the strain for whole-genome sequencing (WGS); hence, 16S rRNA PCR was undertaken using a colony of the SS1954.14 isolate as input DNA. The 27F (AGAGTTTGATCCTGGCTCAG) and 1492R (GGTTACCTTGTTACGACTT) primers were used to amplify a 1.3 kB region of the strain. The amplified section was sent for Sanger sequencing using GENEWIZ sequencing services (https://www.genewiz.com/), and the DNA sequence was searched for matches using EZTaxon to assign the genus [13].

DNA extraction, short-read sequencing, genome assembly and annotation were performed by MicrobesNG, as outlined in [14]. Briefly, the isolate was grown to mid-log in BHI broth, and 10 ml of culture was pelleted, washed with PBS and resuspended in DNA/RNA shield (Zymo Research). The cell suspension was lysed in Tris-EDTA buffer with lysozyme, MetaPolyzyme and RNase A before the addition of proteinase K and SDS. The DNA was purified using solid-phase reversible immobilization beads and resuspended in elution buffer. Library preparation used the Nextera XT library prep kit, and sequencing was undertaken using an Illumina NovaSeq 6000 for short-read sequencing using a 250 bp paired-end protocol. For data processing, the reads were trimmed with Trimmomatic v.0.30 [15], with a sliding-window cut-off of Q15. Assembly was undertaken using SPAdes v.3.7 [16], and the genome annotated using NCBI’s Prokaryotic Genome Annotation pipeline v.6.8 [17].

### Genome analysis

Preliminary species assignment was undertaken by comparing the *gyrB* (DNA gyrase subunit B) nucleotide sequences of reference strains of *Pseudomonas* spp. taken from [18]. Sequence alignment was undertaken using Clustal OMEGA [19]. A maximum-likelihood tree was inferred using IQ-TREE v.1.6.1 [20], with 1,000 bootstraps. The TIM2+F+R3 model was selected using ModelFinder [21], and the resulting phylogenetic tree was displayed using iTOL [22].

The genome was assessed for completeness using CheckM v.1.1.2 with the lineage_wf workflow [23] to confirm that the standards for a high-quality draft genome were met [24]. The species was further investigated using the Genome Taxonomy Database toolkit v. 2.1.1 [25] on the assembled genome using the classify_wf workflow. Average nucleotide identity (ANI) analysis was undertaken using JSpeciesWS, with blast to obtain the ANIb values [26]. Based on the closest matched species being part of the *Pseudomonas koreensis* subgroup, the WGSs of all 23 representative species were downloaded from NCBI, alongside a *Pseudomonas aeruginosa* WGS as an outgroup [27] (Table S1, available in the online Supplementary Material). The genomes were annotated using Prokka v1.11 [28]. The core-genome alignment across all strains was undertaken using the aligner MAFFT within Panaroo v.1.6.0 [29], using strict mode, and variant sites were extracted using SNP-sites v.2.1.5 [30]. A maximum-likelihood tree was inferred using IQ-TREE v.1.6.1 [20], with 1,000 bootstraps. The TIM2+F+ASC+R4 model was selected using ModelFinder [21]. The phylogenetic tree figure was visualized using iTOL [22].

The assembled genome was analysed using antiSMASH v8.0 [31] and BAGEL4 [32]. For antiSMASH, the known matches of biosynthetic gene clusters (BGCs) to the MiBIG database were manually evaluated to assess the likelihood of each compound being encoded. For BAGEL4 outputs, the predicted bacteriocins were compared with the BACTIBASE database [33].

### Hydrogen cyanide assay

This assay, adapted from [34, 35], tested for hydrogen cyanide (HCN) production in three different conditions. (1) SS1954.14 streaked onto BHI agar; (2) 5 µl SS1954.14 (adjusted to 0.5 McFarland turbidity standards) added to 100 µl BHI broth in a 96-well plate; (3) 100 µl CFS, derived from an overnight culture of SS1954.14, added to a 96-well plate. The plates were overlaid with Feigl–Anger reagent paper [36], prepared as outlined. Copper (II) ethyl acetoacetate (5 mg ml^−1^) and 4,4'-methylenebis (*N,N*-dimethylaniline) (5 mg ml^−1^) were dissolved in chloroform. Whatman 3 MM chromatography paper was soaked in the chloroform solution. After complete solvent evaporation, the paper was placed under the plate lids and incubated at room temperature in a fume cupboard for 48 h. The colour change from light green to blue was visually inspected to indicate HCN production, as a binary: production or no production outcome.

### Extraction of unknown natural product

SS1954.14 colonies were grown on BHI agar, subsequently picked and inoculated into 10 ml BHI broth and grown overnight (16–20 h) at 180 r.p.m., 37 °C. The 10 ml BHI broth culture was added to an additional 400 ml of BHI broth and incubated at 180 r.p.m., 37 °C for a further 24 h. The culture was centrifuged at 2,630 ***g*** for 20 min at room temperature, supernatant collected and pellet discarded. A crude extract was obtained from the supernatant via three consecutive extractions using ethyl acetate in a 1 : 1 ratio (v/v). To obtain clear partitioning of the layers during solvent extraction, 5 g of sodium chloride was added. Residual water was removed from the ethyl acetate by passing it through sodium sulphate packed inside a funnel with Whatman I filter paper. The solvent was removed under reduced pressure using a rotary evaporator with the water bath set at 40 °C. The crude product was purified by preparative high-performance liquid chromatography (HPLC) using water and acetonitrile, with an elution gradient of 5 to 25% acetonitrile across 180 min, then isocratic at 25% acetonitrile for 20 min. Column: Agilent PLRP-S (reverse phase); 8 µm, 100 Å, 25 mm inner diameter. Injection vol: 500 µl×3. Flow rate: 6 ml min^−1^. UV signals detected: 210 nm and 280 nm at room temperature.

### Mass spectrometry

High-resolution mass spectra (HRMS) were obtained using an Agilent QTOF 6500 series, equipped with an electrospray interface and operated in positive or negative ion mode.

### HPLC analysis

Elution profiles were compared using an Agilent 1260 infinity analytical HPLC system equipped with a quaternary pump, standard autosampler and multiple-wavelength detector. The HPLC method was undertaken at 25 ℃, using solvents of water containing 0.1% formic acid and acetonitrile containing 0.1% formic acid. The run was isocratic at 5% acetonitrile for 1 min, then had a gradient from 5 to 95% acetonitrile across 11 min and then isocratic at 95% acetonitrile for 4 min. Column: ZORBAX Eclipse Plus C18 (reverse phase); 3.5 µm, 4.6 mm×100 mm. Injection vol: 20 µl for the isolated compound and 10 µl for the commercial maculosin compound. Flow rate: 1.0 ml min^−1^. UV signal detected at 210 nm.

### Minimum inhibitory concentration assay

*C. albicans* was inoculated in potato dextrose broth (PDB), whereas *E. coli* was inoculated into BHI broth. Both sets were incubated for 16–20 h at 180 r.p.m., 37 °C. For *C. albicans*, PDB was used throughout and for *E. coli*, BHI broth was used. The overnight culture was diluted by adding 25 µl to 5 ml fresh broth and vortexed. The OD was adjusted to a 0.5 McFarland standard (OD_600_ 0.095–0.1) and diluted 1 : 50 in broth (i.e. 100 µl diluted culture to 4.9 ml broth). This suspension was further diluted 1 : 1 in broth (i.e. 2.5 ml diluted culture to 2.5 ml broth) and vortexed. A 96-well plate was prepared to contain 95 µl per well of selected compounds or fractions, which were serially diluted from 256 to 1 μg ml^−1^ in broth. The diluted test cultures were added to each well at 5 µl per well. The plates were then incubated at 37 °C for ~20 h; if full growth was not observed, the plates were incubated for a further 20 h. Wells containing no visual growth were used to read the MIC.

## RESULTS

### Isolate SS1954.14 demonstrated antimicrobial activity against *C. albicans*

**Fig. 1.**
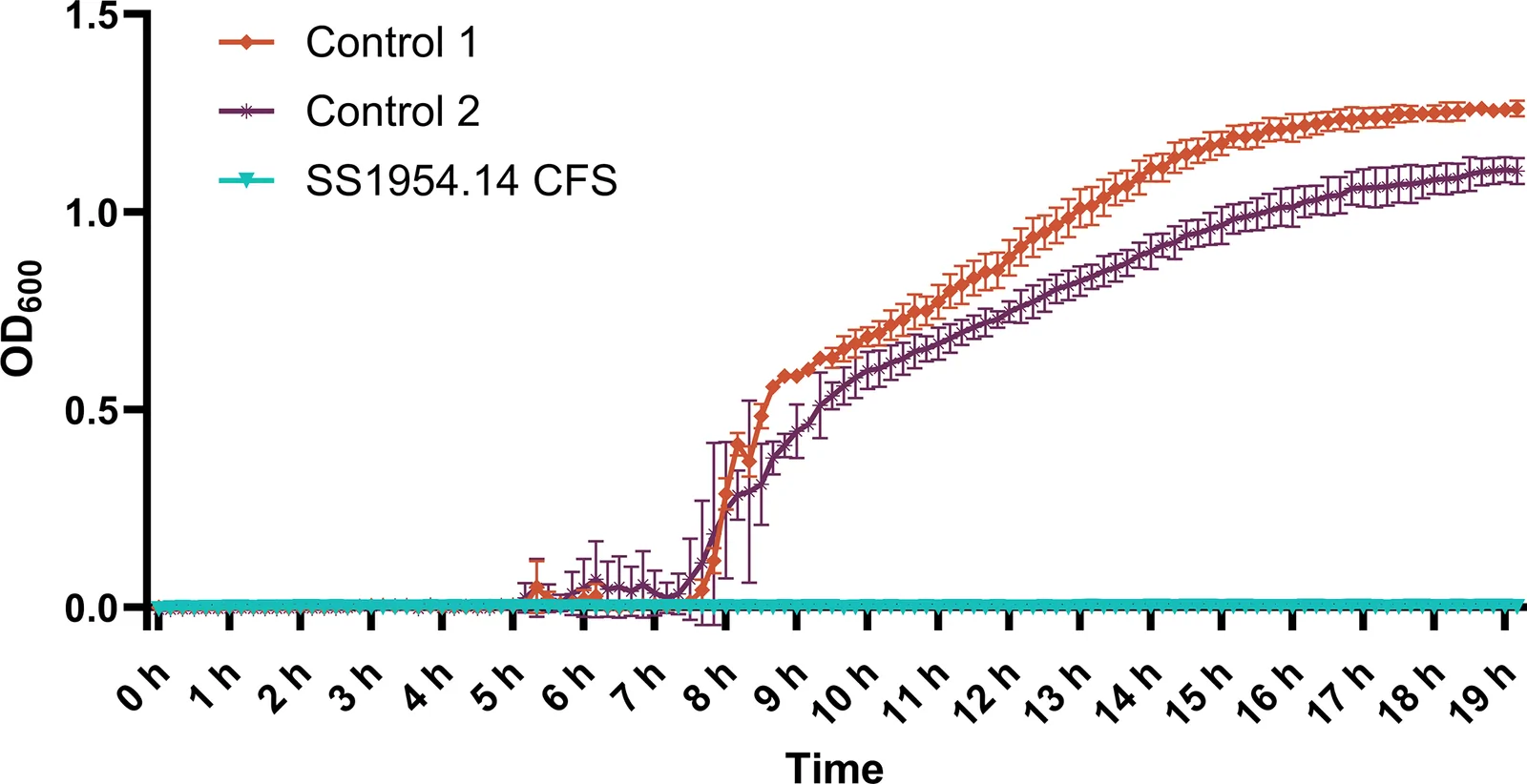
Growth curves of *C. albicans* with and without SS1954.14 CFS. Control 1 is growth of *C. albicans* in fresh BHI (orange), and control 2 is growth of *C. albicans* in *C. albicans* CFS (purple), allowing for comparison.

A beige colony was isolated from a swab taken from a Winogradsky column and designated as strain SS1954.14. In an agar plate-based screening assay, the isolate inhibited the growth of *C. albicans* ATCC 90028 and demonstrated a weaker impact on growth against *E. coli* NCTC 86 (Fig. S1). Given the promising growth inhibition shown by isolate SS1954.14 on agar, we chose to investigate this further by looking at antimicrobial production when the isolate was grown alone in broth. Following 20 h of growth in BHI broth, we tested the CFS of this isolate for its ability to inhibit the growth of *C. albicans* (Fig. 1). In the CFS assay, the strain exhibited strong antimicrobial activity against *C. albicans*, resulting in a 98.82% reduction in area under the curve over 19 h relative to the control (*C. albicans* grown in *C. albicans* CFS).

### WGS of SS1954.14 suggested it was a *P. monsensis* strain

The strain was identified as a *Pseudomonas* sp. through comparison of the 16S rRNA gene sequence using the EZTaxon server database [13]. The strain was then WGS using short-read sequencing. The assembled genome was 6.36 Mb in length, with a GC content of 60.13%. The genome had a coverage of 37.7×, and the N50 of the genome was 504,156 bp. The assembled genome contained 66 contigs and 5,575 predicted genes, including four complete rRNAs and 63 tRNAs. The completeness of the genome was 99.39% with 0.32% contamination, using the *Pseudomonas* CheckM marker set.

**Fig. 2.**
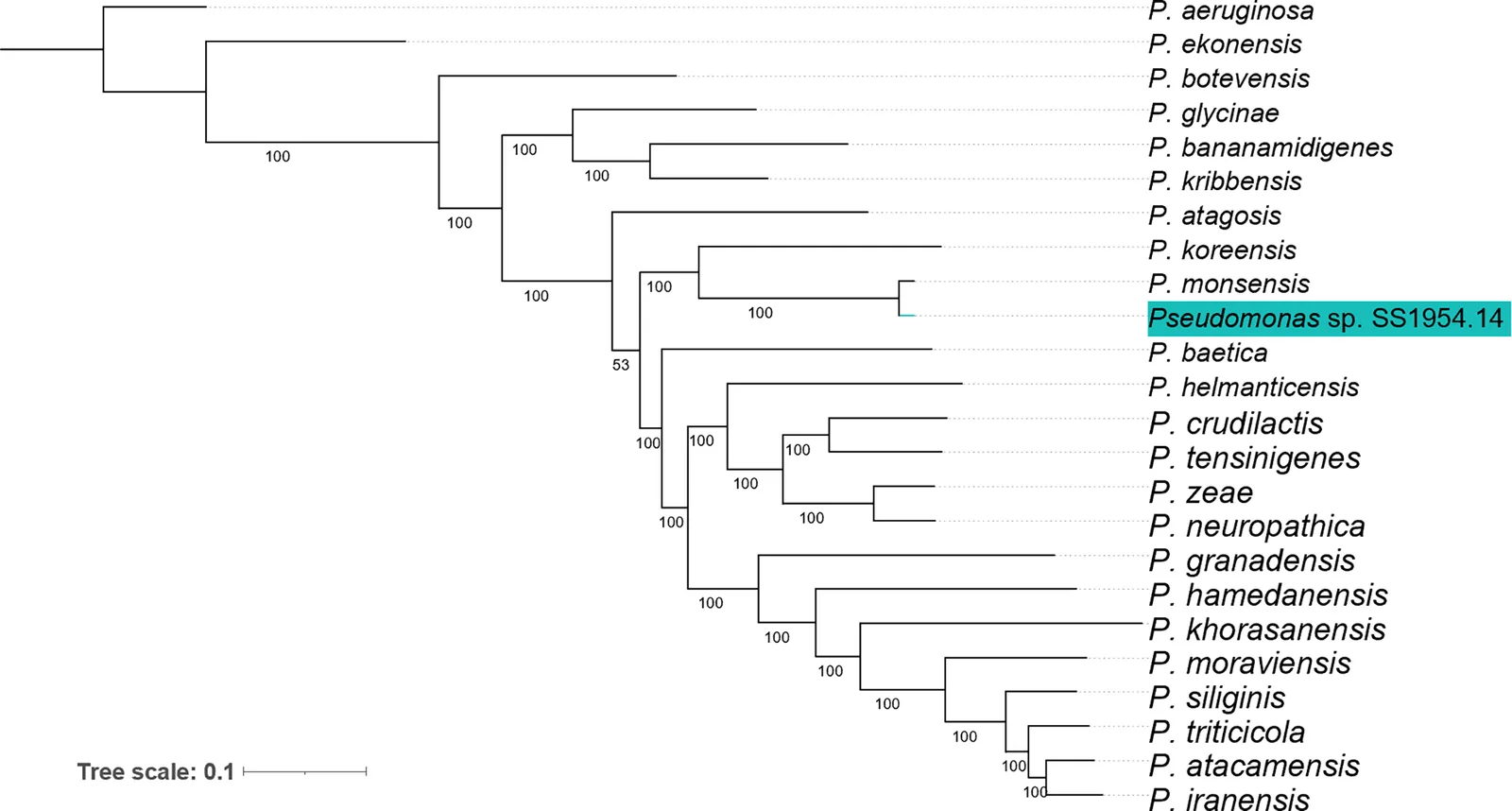
Phylogenetic tree based on SNPs present within a core-genome alignment, comparing all *P. koreensis* subgroup species with SS1954.14 (highlighted in teal). *P. aeruginosa* was used as the outgroup. Type-strain information is provided in Table S1. The tree was created using IQ-TREE, with 1,000 bootstrap replicates performed and displayed using iTOL [20, 22]. The tree scale represents SNPs per site within the core-genome alignment.

The *gyrB* (encoding DNA gyrase subunit B) gene was taken from the genome and compared with other *gyrB* sequences found to be part of different multilocus sequence analysis groups of the *Pseudomonas fluorescens* complex [18]. The most similar *gyrB* sequences were found in the *P. koreensis* strains included in the analysis (Fig. S2). The closest match was to *P. koreensis* LMG 21318 [37], and this strain had an ANI of 88.53 (77.22% coverage) to our *Pseudomonas* sp*.* SS1954.14 strain’s genome. However, due to the low ANI value/percentage coverage, we reassessed the species identification based on the WGS against the Genome Taxonomy Database [25]. This identified *P. monsensis* LMG 32033 as the closest match, with an ANI of 96.38 (95% coverage) calculated by the Genome Taxonomy Database toolkit or an ANIb of 98.55 (93.54% coverage) calculated by JSpeciesWS [25–27]. *P. koreensis* A9 was the second-closest match, with an ANI of 90.3 (95% coverage). *P. monsensis* has recently been included in the *P. koreensis* subgroup based on ANI comparisons across all *Pseudomonas* spp. [27]. The WGSs of all 23 species grouped within the *P. koreensis* subgroup were compared based on a core-genome alignment and by comparing single-nucleotide polymorphisms (SNPs) that were present within these core genes (Fig. 2). This highlighted that *Pseudomonas* sp. SS1954.14 grouped most closely with the *P. monsensis* type strain, in support of ANI-based identification of *Pseudomonas* sp. SS1954.14 belonging to the *P. monsensis* species.

### Genome analysis indicated several possible antimicrobial compounds

Given the initial anti-*Candida* activity of the isolate, we analysed the genome for BGCs using antiSMASH 8.0 (Table 1) [38]. Only two BGCs had high-similarity matches: those predicted to encode HCN and lokisin. Although HCN production has been linked to suppression of fungal growth [35, 39], this has primarily been on agar, and it is unlikely to contribute to the CFS activity observed against *C. albicans*. The non-ribosomal peptide synthetase (NRPS) BGC identified in region 5.3 had 100% similarity matches listed in antiSMASH to several BGCs (defined as all genes in the NRPS region as >45% amino acid identity across >40% coverage [40]), including lokisin, anikasin, viscosinamide A, bananamide 1, putisolvin and gacamide A. Hence, the NRPS was manually checked to confirm that it was predicted to encode the Leu-Asp-Thr-Leu-Leu-Ser-Leu-Ser-Leu-Ile-Asp amino acid sequence, which represents the core structure of lokisin. The three NRPS genes in region 5.3 were most similar to the lokisin BGCs, with each pair of genes having 90% amino acid identity (99% coverage), 91% identity (100% coverage) and 88% identity (100% coverage) for ctg5_338, 339 and 340, respectively.

**Table 1.** Analysis of predicted BGCs using antiSMASH 8.0. If no similar BGCs were present in the MiBIG database, this column was left blank

Region	Type of BGC	Position on contig	Most similar BGC in MiBIG	Similarity confidence
1.1	NRP metallophore	94,600–178,063	Pf-5, pyoverdine	Low
1.2	RiPP-like	425,019–435,858		N/A
2.1	RiPP-like	168,445–179,290		N/A
5.1	β-Lactone	42,739–66,001	Fengycin	N/A
5.2	Hydrogen cyanide	192,619–205,591	HCN	High
5.3	NRPS	349,953–427,417	Lokisin	High
5.4	RiPP-like	511,538–523,742		N/A
6.1	NRPS	16,420–69,418	Pf-5 pyoverdine	Low
6.2	NAGGN	210,452–225,332		N/A
7.1	RiPP-like	44,312–55,199		N/A
8.1	HCN	279,654–292,471		N/A
10.1	Terpene-precursor	246,130–267,017		N/A
10.2	RRE-containing	285,582–306,715		N/A
12.1	Redox-cofactor	77,830–99,986	Lankacidin C	N/A
19.1	NRPS-like	1–17,407		N/A

The lower-percentage similarity matches included pyoverdine, fengycin and lankacidin C. The pyoverdine BGC appears to have been split across several contigs during the genome assembly process, suggesting repetitive DNA sequences internal to the 100 kbp genome region [41]. The weak match to fengycin was based on two genes with 48% nucleotide identity (68 and 95% coverage) located upstream of the five main biosynthetic genes encoding proteins involved in fengycin production [42, 43]. Hence, this cluster is unlikely to encode fengycin in *Pseudomonas* sp. SS1954.14. The low-similarity match in region 12.1 was matched to lankacidin C, on the basis of two genes with 46 and 49% nucleotide identity (88 and 89% coverage). However, the matching genes appear to encode pyrroloquinoline quinone biosynthesis proteins C and E. These are part of a five-gene BGC encoding pyrroloquinoline quinone [44], which is adjacent to the lankacidin C BGC found in *Streptomyces rochei* [45].

We also analysed the genome for bacteriocins and other antimicrobial peptides using BAGEL4 [32]. This analysis identified two regions of interest on contigs 4 and 16, neither of which had been highlighted by antiSMASH (Table 2). These encode putative colicin-like bacteriocins, which are a class of antimicrobial peptides produced by bacteria. Both genes were directly followed by genes encoding colicin immunity proteins, which likely provide self-immunity from the toxin.

**Table 2.** Analysis of predicted bacteriocins and RiPPs using BAGEL4. The most similar bacteriocins were identified using BACTIBASE

Region	Position on contig	Class	Most similar bacteriocin	% amino acid identity (% coverage)
4	245,639–266,836	Colicin-like bacteriocin	Carocin D	21 (58)
16	14,945–35,332	Colicin-like bacteriocin	Pyocin S1	20 (42)

Once the putative bacteriocins had been identified, the translated sequences of the predicted genes were compared against the BACTIBASE database of bacteriocins [33]. These were compared using BLASTp against the bacteriocins database and found two low amino acid identity matches (Table 2). These bacteriocins were identified in *Pectobacterium carotovorum* subsp. *carotovorum* for carocin D and *P. aeruginosa* for pyocin S1 [46, 47].

### HCN was not detected in the CFS of SS1954.14

As the SS1954.14 strain encoded BGCs linked to the production of HCN, we performed a detection assay using Feigl–Anger reagent paper. It was observed that the strain produced HCN when grown on BHI agar and in BHI broth, but there was no detection of HCN when the reagent paper was placed above wells containing only CFS derived from SS1954.14 (Fig. S3). For the antimicrobial activity experiments and downstream extractions, CFS of SS1954.14 was used, and hence it is unlikely HCN was responsible for the antimicrobial effect against *C. albicans*.

### Preparative HPLC fractionation identified maculosin as the active compound

**Fig. 3.**
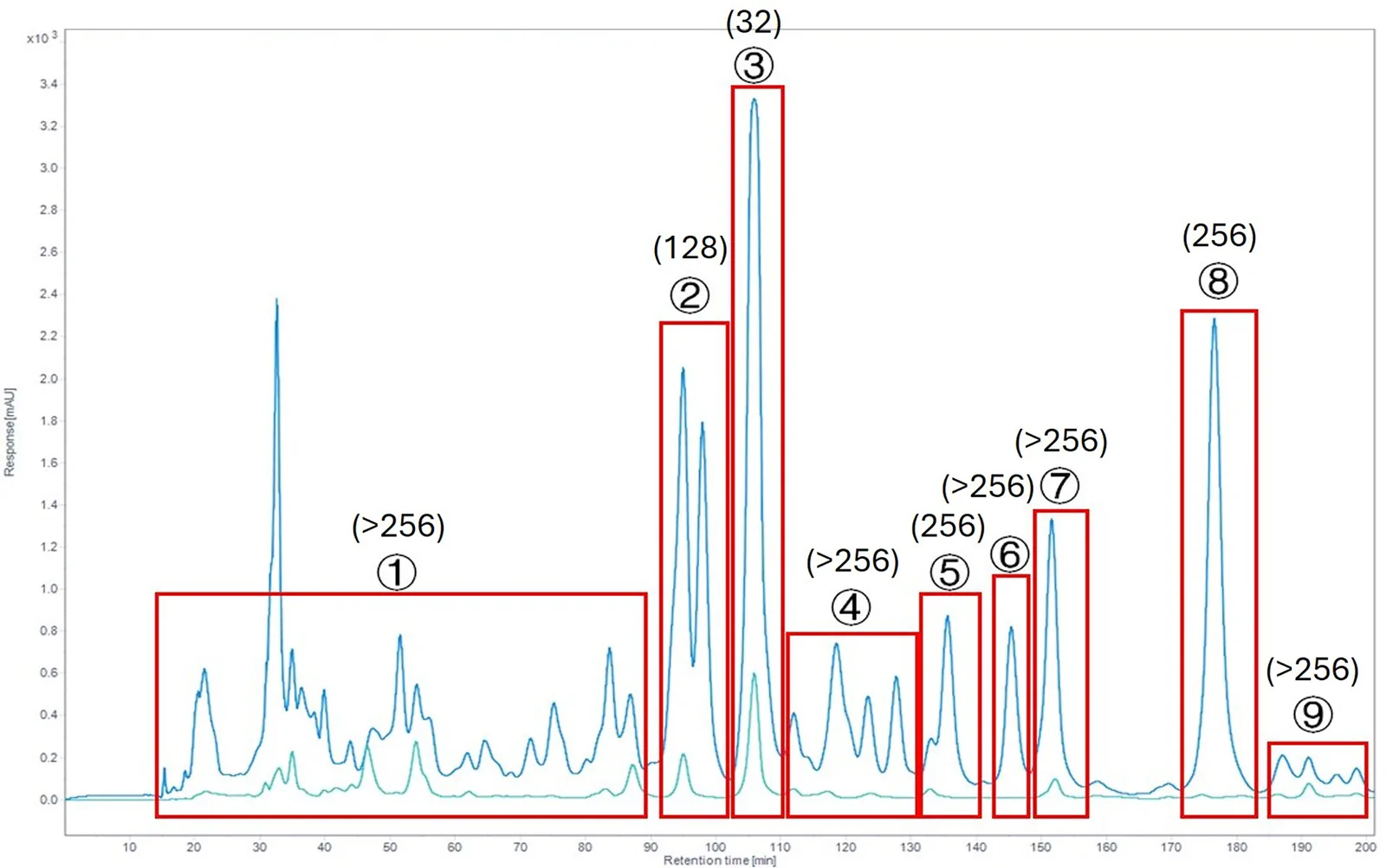
Chromatogram of the HPLC separation and fractionation of SS1954.14 ethyl acetate-extracted CFS, highlighting the nine groups of fractions collected. Above each fraction number is the minimum inhibitory concentration (MIC) of the fraction against *C. albicans* ATCC 90028 in µg ml^−1^. Overlaid are the detection at 210 nm (blue) and 280 nm (green).

Ethyl acetate extraction of the CFS from *Pseudomonas* sp. SS1954.14 was performed to remove growth media and other impurities. The extract was further purified via preparative HPLC using an elution gradient of 5 to 25% acetonitrile. All nine fractions were collected and screened for antifungal and antibacterial activity to identify the most bioactive compounds within the sample (Fig. 3).

Fraction 3 was found to show the highest purity and exhibit the strongest inhibition activity, with the lowest minimum inhibitory concentration (MIC) recorded at 32 µg ml^−1^ against *C. albicans* (Fig. 3). No activity was observed for any of the fractions when tested against *E. coli* (MIC at >256 µg ml^−1^).

**Fig. 4.**
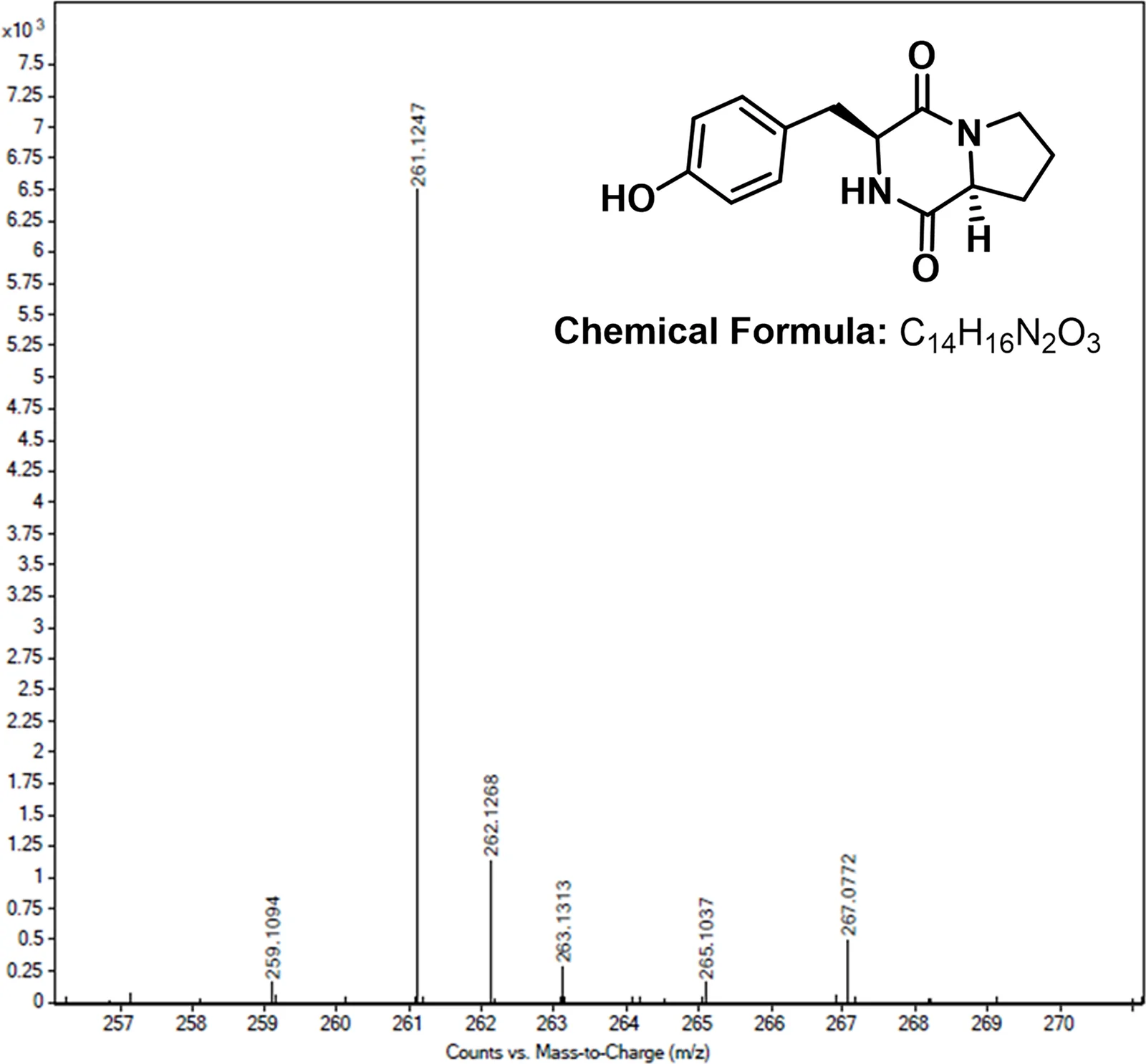
HRMS identifying peak at 261.1247 Da (positive -ion ESI-MS, (m/z)) and corresponding cyclo-(Pro-Tyr) structure.

As fraction 3 had the strongest activity against *C. albicans*, we selected this fraction for downstream evaluation. We performed HRMS on this fraction and identified several peaks (Fig. S4). One of the major peaks found was at 261.1247 Da (positive-ion ESI-MS, m/z) (Fig. 4). This matched the reported masses of 261.1241 Da [48] and 261.124 Da [49] found to correspond to cyclo(Pro-Tyr), also known as maculosin. We purchased maculosin from a commercial source (Bachem) and repeated the MIC testing. This gave the same MIC of 32 µg ml^−1^ against *C. albicans* that was obtained for fraction 3. In addition, we compared the HPLC elution profile of the extracted natural product to the commercial maculosin compound (Fig. S5). The two compounds had consistent retention times and identical chromatographic behaviour.

## Discussion

A strain of *P. monsensis* has been identified that displayed antifungal activity against *C. albicans,* and we confirmed that this strain likely produced maculosin. Within the reported genome, we have also identified several BGCs, but none of them has been associated with maculosin biosynthesis.

Maculosin has been found in the CFS of *Pseudomonas* spp. and has previously been reported to have anti-quorum-sensing activity [50, 51]. The production of maculosin was also found to be negatively regulated by the LasI quorum-sensing system in *P. aeruginosa* [52]. The exact route of maculosin biosynthesis in known bacterial producers has not been identified yet. Production could be through a cyclodipeptide synthase enzyme, of which an expressed enzyme from *Diplorickettsia massiliensis* 20B was shown to produce cyclo(l-Pro-l-Tyr) 23% of the time [53]. However, this is the only report of a cyclo(l-Pro-l-Tyr)-producing cyclodipeptide synthase that we could identify, with an overall low frequency of this type of cyclodipeptide being associated with the tRNA-dependent enzymes [54]. We searched the *Pseudomonas* sp. SS1954.14 genome sequence using the nucleotide sequences corresponding to the characterized cyclodipeptide synthase protein from *D. massiliensis* 20B and from other *Pseudomonas* spp. that produced cyclo(l-Glu-l-Ala) and cyclo(l-Leu-l-Glu) [53, 54]. However, no matches were identified. The production of maculosin has been shown to be affected by gene deletions in other *Pseudomonas* BGCs [51]. This may suggest that there is an alternative pathway for synthesis, which is the case for several other cyclodipeptides containing either tyrosine or proline [51, 54]. The other pathway for synthesis of cyclodipeptides is NRPS BGCs [55], of which only the NRPS on contig 19 of the *Pseudomonas* sp. SS1954.14 genome is not associated with a known compound. The NRPS gene present at 2,970–6,362 bp had a similarity score of 0.61 to the peramine BGC producing an l-Pro, l-Arg dipeptide [56], and of 0.59 to the fungisporin BGC producing a cyclic tetrapeptide of d-Phe-l-Phe-d-Val-l-Val [57]. However, the contig 19 NRPS is shorter and predicted to only add one aliphatic, branched, hydrophobic amino acid to a compound, with the protein amino acid identity of 28.4% (72% coverage) to the peramine NRPS gene and 26.9% (52% coverage) to the fungisporin NRPS gene. To investigate the role of the *P. monsensis* BGCs in the synthesis of maculosin, knockout mutations within individual BGC genes could be produced to observe loss of compound production.

Maculosin has previously been found to have antifungal activity against *Aspergillus flavus*, *C. albicans*, *Colletotrichum truncatum*, *Fusarium oxysporum*, *Penicillium expansum* and *Rhizoctonia solani* [58–60]. Against *C. albicans* specifically, it has been found to have MICs of 27 µg ml^−1^ [59] and 32 µg ml^−1^ [58], but another group reported no activity, suggesting putative inhibition would require >256 µg ml^−1^ [61]. Finally, a further group reported no zone of clearance following addition of 500 µg ml^−1^ to a sterile disc [62]. All four results were derived using different testing methods, preventing direct comparisons, with two papers not reporting the growth media used in either their broth microdilution assay [59] or their disc diffusion assay [62]. In addition, the strains of *C. albicans* used were different, being *C. albicans* ATCC2091 [58], *C. albicans* ATCC MYA-2876 [61] and two unspecified *C. albicans* spp. [59, 62]. The most comparable method to ours used broth microdilution in PDB and found the same MIC of 32 µg ml^−1^ [58]. The other broth microdilution approach used yeast nitrogen base broth and found an MIC of >256 µg ml^−1^ [61]. As both media have similar pH, this suggests that the difference in MIC was unlikely to be due to media acidity. This may indicate that the *C. albicans* ATCC MYA-2876 genome encodes resistance to maculosin that is not present in *C. albicans* ATCC 90028 or ATCC 2091.

The exact stereoisomer of cyclo(Pro-Tyr) present within our extracted fraction cannot be confirmed based on the mass spectrometry match, which would be identical for the four possible configurations: cyclo(l-Pro-l-Tyr), cyclo(d-Pro-l-Tyr), cyclo(l-Pro-d-Tyr) and cyclo(d-Pro-d-Tyr). In addition to maculosin, we found references only to the activity of cyclo(l-Pro-d-Tyr) against *C. albicans*, with reported MICs of 32 and 51.25 µg ml^−1^ [58, 63]. We suspect our extracted compound is maculosin [cylco(l-Pro-l-Tyr)], as only the ll form has previously been isolated from *Pseudomonas* spp.; no epimerization domains (associated with d-amino acids) were present in unassigned BGCs in the genome; and the HPLC elution profile and MIC were identical to commercially obtained maculosin (Fig. S5). Future work will undertake supercritical fluid chromatography separation to compare our extracted compound with other stereoisomers to confirm the identity and explore their activity against *C. albicans*.

Given the limited number of approved antifungal compounds in clinical use and only a handful of new compounds within the pipeline, the development of additional antifungal compounds is needed. Testing maculosin or its analogues against drug-resistant *C. albicans* isolates could provide greater incentive for its continued development, given the increasing levels of drug resistance. This study highlights that maculosin, as a bacterial natural product, may offer a potential antifungal compound requiring further drug development. Our work also shows that a public outreach project can engage participants and enthuse future scientists about antimicrobial drug discovery [12], while also investigating antimicrobial compounds.

## Supplementary material

10.1099/acmi.0.001229.v3 Supplementary Material 1.

## Abbreviations

ANI: average nucleotide identity
BGC: biosynthetic gene cluster
BHI: brain heart infusion
CFS: cell-free supernatant
HCN: hydrogen cyanide
HPLC: high-performance liquid chromatography
HRMS: high-resolution mass spectrometry
NRPS: non-ribosomal peptide synthetase
PDB: potato dextrose broth
SNP: single-nucleotide polymorphism
WGS: whole-genome sequencing
