# Supplementary material for: Analysis of the genome of a Pseudomonas monsensis isolate that produces the antifungal dipeptide maculosin
Source: Access Microbiol. 2026 Sep 15;8(9):001229.v3. doi: 10.1099/acmi.0.001229.v3 (PMC13577705; doi:10.1099/acmi.0.001229.v3)
Supplement: Supplementary file 1 [file acmi-8-01229-s001.pdf]

Supplementary Material for 'Analysis of the genome of a *Pseudomonas monsensis* isolate which produces the anti-fungal dipeptide maculosin'

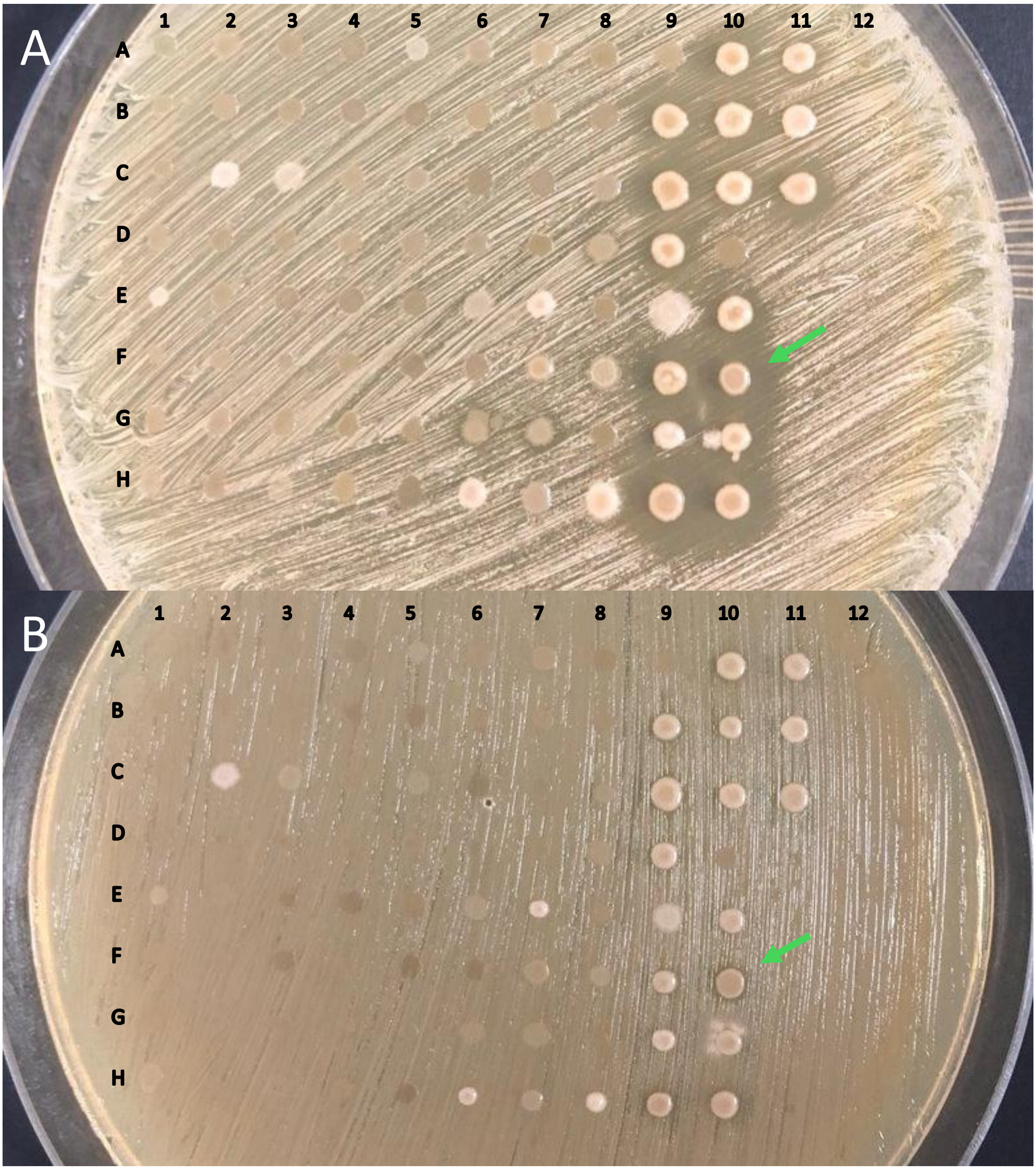

Supplementary Figure S1: Screening of Swab and Send plate SAS211, with SS1954.14 being screened in position 10F. Isolate is indicated with an arrow. (a) Screening on top of a lawn of *C. albicans*. (b) Screening on top of a lawn of *E. coli*.

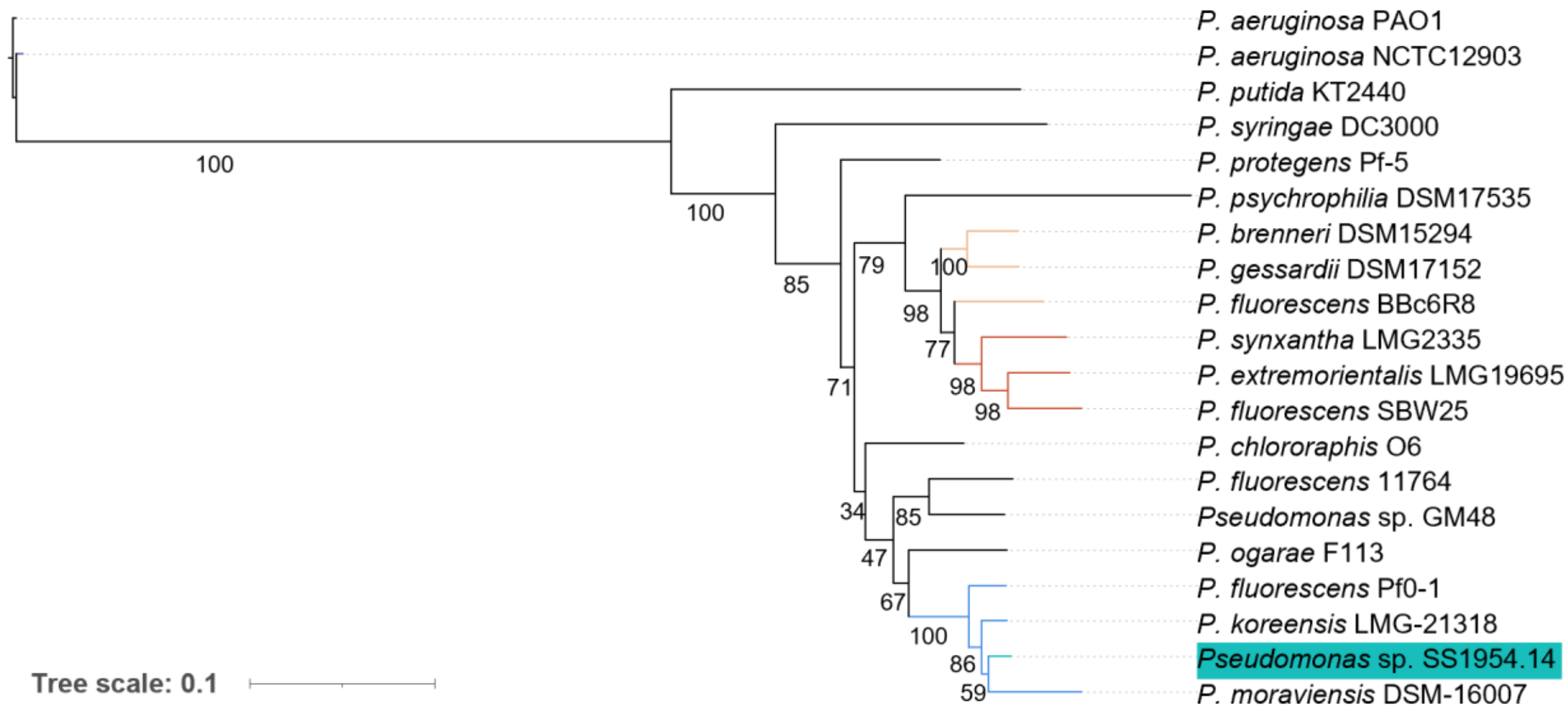

Supplementary Figure S2: Phylogenetic tree using the *gyrB* sequences of *Pseudomonas* spp. to assign the most likely species of SS1954.14 (highlighted in teal). Branches are coloured if more than one species from the multi-locus sequence analysis group of *Pseudomonas* is present. This includes *P. koreensis* in blue, *P. aeruginosa* in purple, *P. fluorescens* in orange and *P. gessardii* in beige. Several strains are labelled as *P. fluorescens,* as they are part of the larger *P. fluorescens* complex but group more closely with other species of *Pseudomonas*, as outlined in [18]. Figure produced using Clustal Omega, IQTree and iTOL [19,20,22].

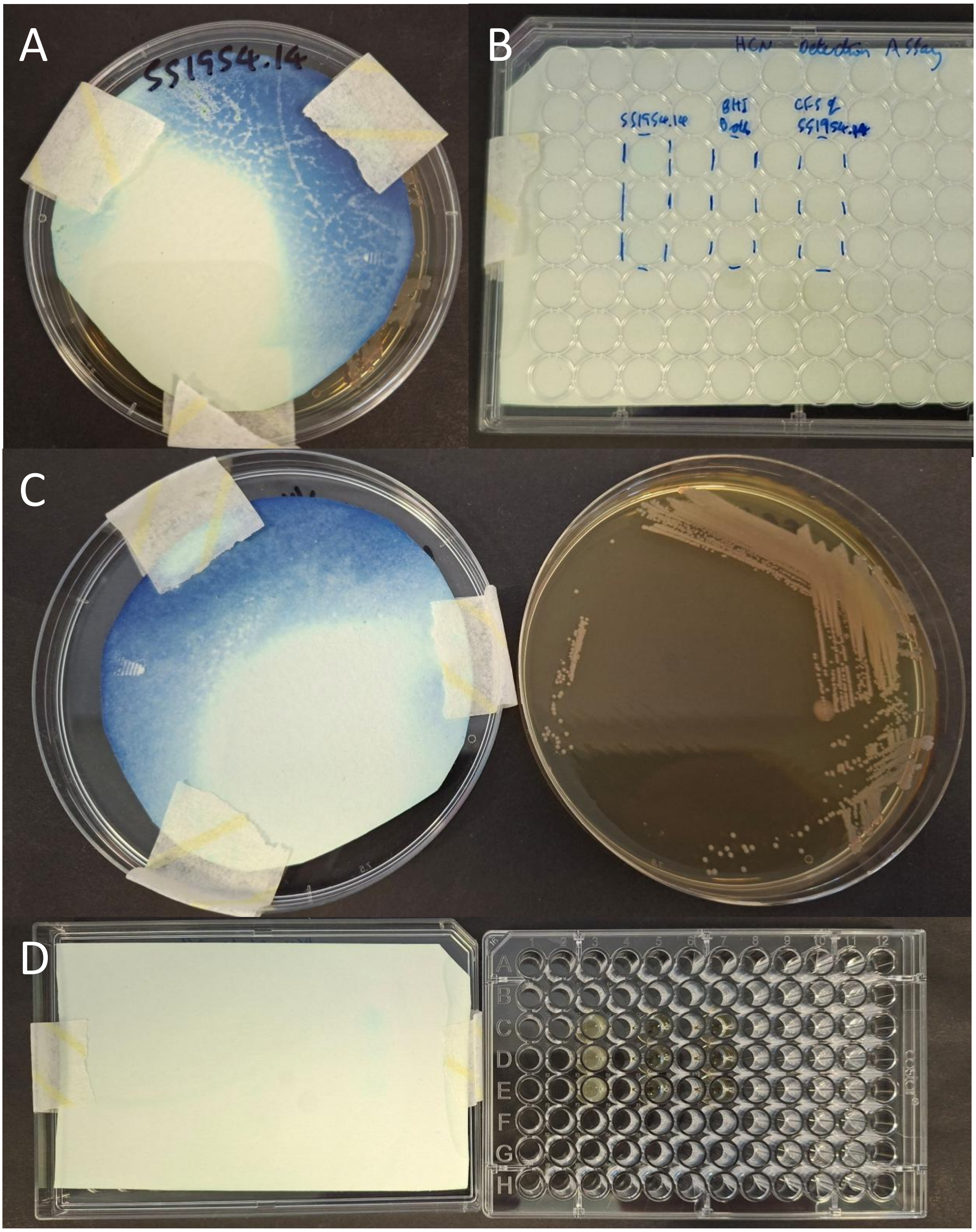

Supplementary Figure S3: HCN detection assay using Feigl–Anger reagent paper placed above and left for 48-hours. (A + C) is growth of SS1954.14 on BHI agar, photographs of the same plate with the lid closed or open. (B +D) is growth of SS1954.14 in BHI broth, or CFS of SS1954.14 in a 96-well plate, photographs of the same plate with the lid closed or open.

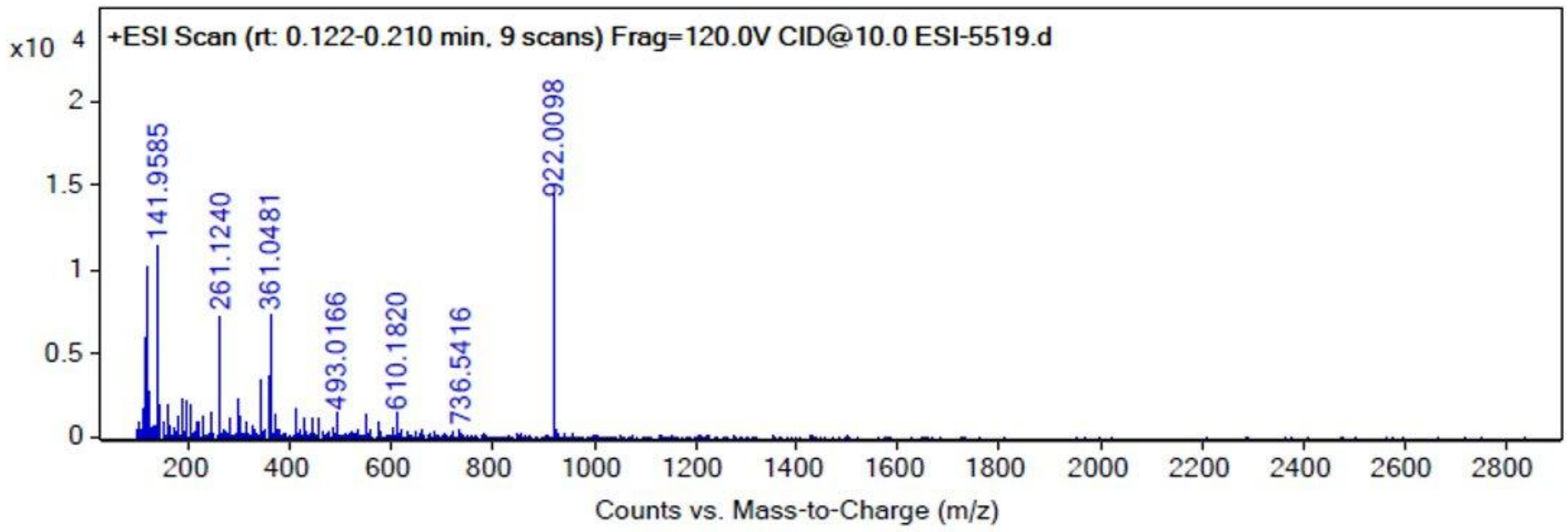

Supplementary Figure S4: ESI+ scan of fraction 3 of the ethyl acetate extract from *Pseudomonas* sp. SS1954.14. The peaks at 141.9585 and 922.0098 m/z are due to spiked in control compounds.

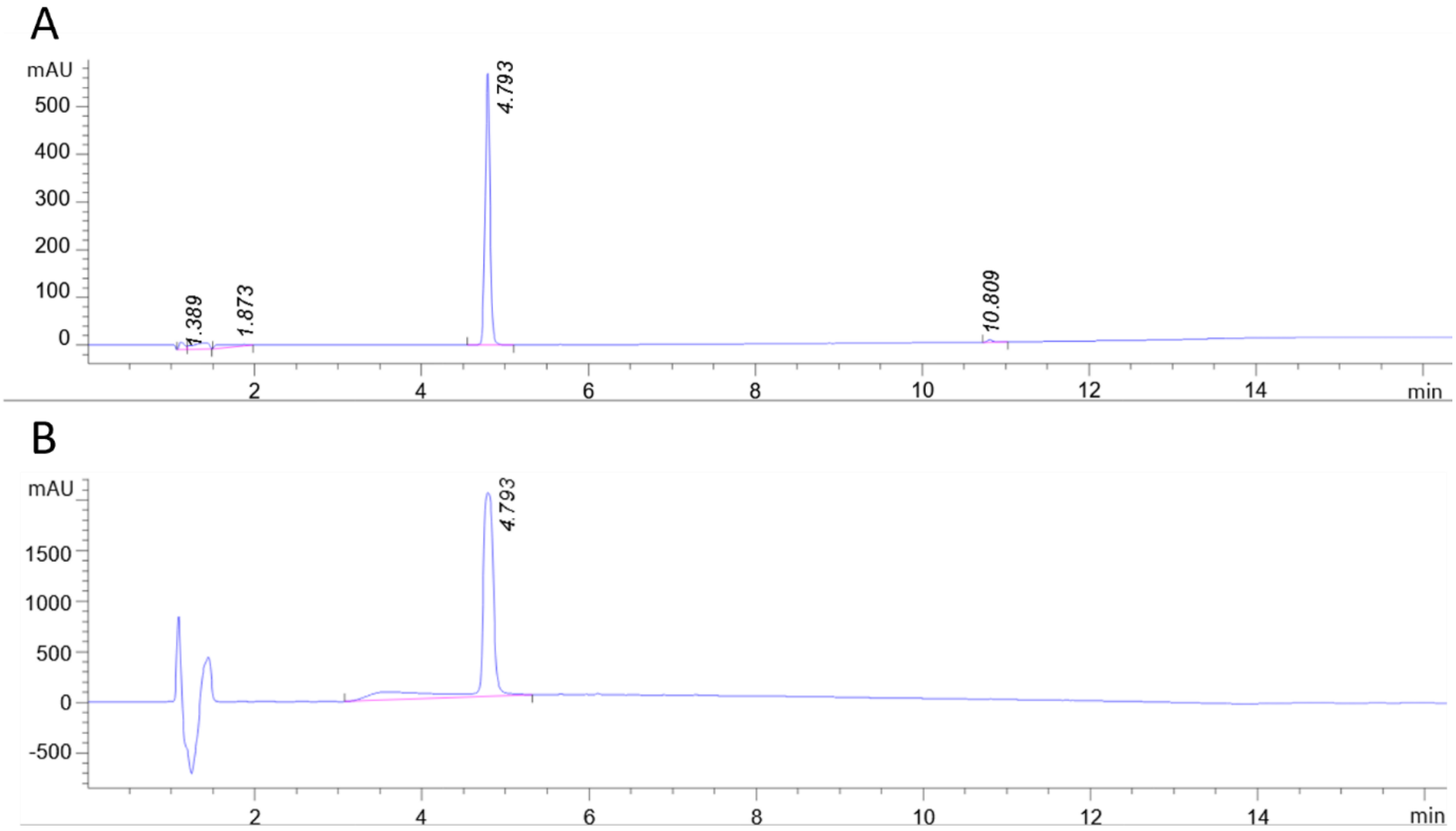

Supplementary Figure S5: Comparison of the elution profiles of the isolated maculosin compound (A) and commercial maculosin compound (B) using HPLC separation. Monitoring absorbance at 210 nm.

Supplementary Table S1: Genome sequence information of the *P. koreensis* sub-group species and *P. aeruginosa*. Species and type strain information taken from [24]. * = IMG genome ID.

**Species**	**Type Strain**	**Accession Number**
Pseudomonas aeruginosa	ATCC 10145 = LMG 1242 = DSM 50071	CP012001.1
Pseudomonas atagosis	PS14 = CECT 9940 = LMG 31496	VXCA00000000.1
Pseudomonas baetica	a390 = CECT 7720	PHHE00000000.1
Pseudomonas crudilactis	UCMA 17988	WXVV00000000.1
Pseudomonas glycinae	MS586 = NRRL B-65441 = LMG 30275	CP014205.2
Pseudomonas koreensis	LMG 21318	LT629687.1
Pseudomonas kribbensis	DSM 100278	CP029608.1
Pseudomonas granadensis	DSM 28040 = LMG 27940	2667527397*
Pseudomonas helmanticensis	LMG 28168 = CECT 8548	2617270757*
Pseudomonas moraviensis	DSM 16007	LT629788.1
Pseudomonas neuropathica	P155 = CECT 30178 = CCUG 74875	JACOPX000000000.1
Pseudomonas atacamensis	SWRI76	SSBS00000000.1
Pseudomonas bananamidigenes	BW11P2	LRUN01000001.1
Pseudomonas botevensis	COW3	JAHTKI000000000
Pseudomonas ekonensis	COR58	JAHSTS000000000
Pseudomonas iranensis	SWRI54	CP077092.1
Pseudomonas khorasanensis	SWRI153	JABWQP000000000
Pseudomonas monsensis	PGSB 8459	CP077087
Pseudomonas tensinigenes	ZA 5.3	CP077089
Pseudomonas hamedanensis	SWRI65	CP077091
Pseudomonas siliginis	SWRI31	CP099598.1
Pseudomonas triticicola	SWRI88	JAHSTX000000000
Pseudomonas zeae	OE 48.2	CP077090
